# Chelation of Mitochondrial Iron as an Antiparasitic Strategy

**DOI:** 10.1021/acsinfecdis.3c00529

**Published:** 2024-01-30

**Authors:** Dominik Arbon, Jan Mach, Aneta Čadková, Anna Sipkova, Jan Stursa, Kristýna Klanicová, Marta Machado, Markus Ganter, Viktoriya Levytska, Daniel Sojka, Jaroslav Truksa, Lukáš Werner, Robert Sutak

**Affiliations:** † Department of Parasitology, Faculty of Science, Charles University, BIOCEV, Vestec 25250, Czech Republic; ‡ Institute of Biotechnology, Czech Academy of Sciences, BIOCEV, Vestec 25250, Czech Republic; § Laboratory of Clinical Pathophysiology, Diabetes Centre, Institute for Clinical and Experimental Medicine, Videnska 1958/9, 140 21 Prague, Czech Republic; ∥ Department of Organic Chemistry, Faculty of Science, Charles University, Prague 128 00, Czech Republic; ⊥ Graduate Program in Areas of Basic and Applied Biology, Instituto de Ciências Biomédicas Abel Salazar, Universidade do Porto, Porto 4050-313, Portugal; # Centre for Infectious Diseases, Parasitology, Heidelberg University Hospital, Heidelberg 69120, Germany; ¶ Institute of Parasitology, Biology Centre, Academy of Sciences of the Czech Republic, Branišovská 1160/31, České Budějovice 37005, Czech Republic

**Keywords:** iron, chelation, parasites, protists, mitochondria

## Abstract

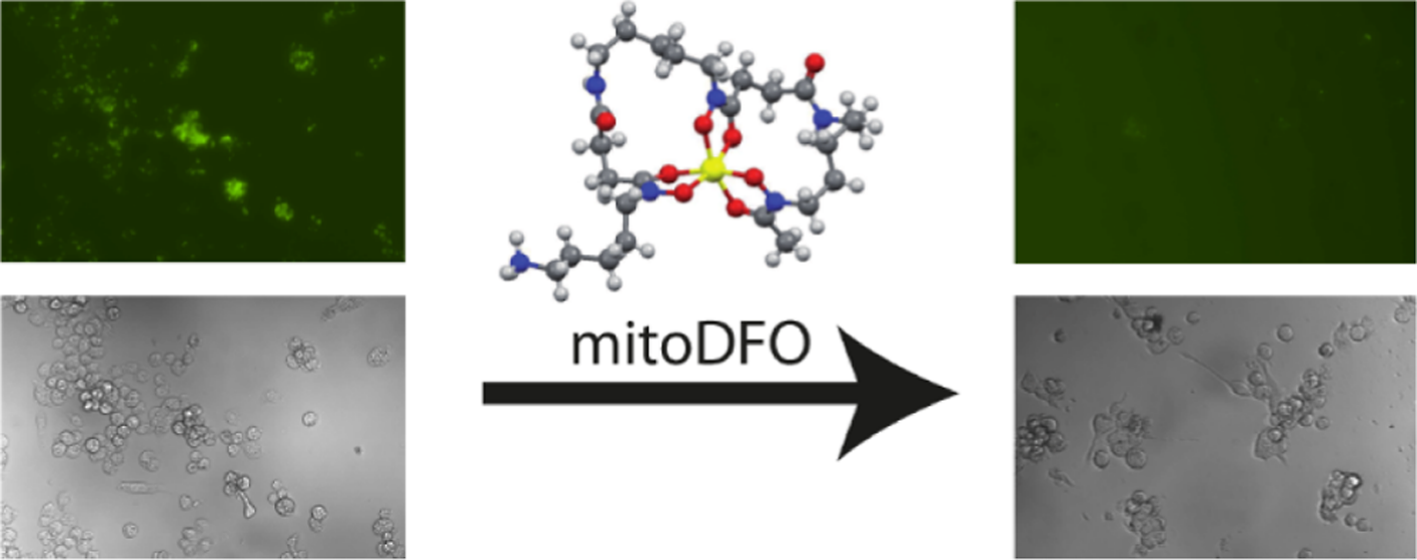

Iron, as an essential micronutrient, plays a crucial role in host–pathogen interactions. In order to limit the growth of the pathogen, a common strategy of innate immunity includes withdrawing available iron to interfere with the cellular processes of the microorganism. Against that, unicellular parasites have developed powerful strategies to scavenge iron, despite the effort of the host. Iron-sequestering compounds, such as the approved and potent chelator deferoxamine (DFO), are considered a viable option for therapeutic intervention. Since iron is heavily utilized in the mitochondrion, targeting iron chelators in this organelle could constitute an effective therapeutic strategy. This work presents mitochondrially targeted DFO, mitoDFO, as a candidate against a range of unicellular parasites with promising in vitro efficiency. Intracellular *Leishmania* infection can be cleared by this compound, and experimentation with *Trypanosoma brucei* 427 elucidates its possible mode of action. The compound not only affects iron homeostasis but also alters the physiochemical properties of the inner mitochondrial membrane, resulting in a loss of function. Furthermore, investigating the virulence factors of pathogenic yeasts confirms that mitoDFO is a viable candidate for therapeutic intervention against a wide spectrum of microbe-associated diseases.

The mitochondrion is a unique organelle that plays a central role in a plethora of biochemical processes. One of its most prominent functions is the ability to generate energy through the tricarboxylic acid cycle and the electron transport chain, coupled with oxidative phosphorylation. An integral part of this process is the generation of an electrochemical gradient (Δψ) across the inner mitochondrial membrane (IMM), and this electrochemical gradient can be exploited as a driving force for specific experimental targeting of this organelle. It is the interplay of sufficiently lipophilic cations, such as triphenylphosphonium (TPP), with Δψ electrostatic gradient that allows the uptake and accumulation of molecules in the mitochondrion.^1,2^ This tool offers a wide range of applications, for example, as an imaging probe, a biochemical marker, and, importantly, as a vector for therapeutics.^3^ The latter use is being explored in anticancer therapy due to the remarkable difference in cancer cell mitochondrial function and the resulting susceptibility to TPP targeting.^4,5^ This phenomenon has led to the discovery of specific mitochondrially targeted anticancer drugs, as in the case of the promising novel drug MitoTam, which has currently passed stage I/Ib clinical trials.^6^ This TPP-tagged Tamoxifen is acting via an altered mode of action and has been shown to be more selective than its untagged counterpart.^7^ Subsequently, MitoTam has been found to be effective against several protozoan parasites, both in vitro and in vivo.^8^

Various derivatives of phosphonium salts have previously been studied as potential therapeutics against different parasitic organisms such as *Leishmania* and *Trypanosoma*, with the common finding that this class of compounds, as expected, affects mitochondrial energy metabolism, resulting in a drop in ATP levels combined with depolarization of mitochondrial membrane potential and possibly mitochondrial disintegration.^8−10^ In addition, F_O_F_1_ ATPase was speculated to be a possible target of this class of compounds.^11^ To their advantage, it has been found that phosphonium salts do not rely on known drug transporters in *Trypanosoma*; therefore, their use as therapeutics could circumvent one common cause of drug-resistant phenotypes.^12^

Although it is well understood that TPP is effective in targeting mitochondria, a rational consideration of the pharmacophore is required to achieve antimicrobial selectivity. Ideally, it should be selected to affect vital processes that take place in the mitochondria, while the targeted pathways are unique to the parasitic organism or more critical to the pathogen than to its host. Iron plays a vital role in countless cellular processes, with the mitochondria being the center of its metabolism and utilization. Iron requirements are particularly high in rapidly dividing pathogens, and their meticulous homeostasis is the basis for one of the strategies of nutritional immunity in mammals.^13^ For this reason, we focused on iron chelation therapy, which has been previously studied as a promising anticancer strategy^14^ and also as a possible antiparasitic intervention.^13,15−17^

Deferoxamine (DFO) is a potent iron chelator with a strong affinity for ferric iron.^18^ Although it has been approved and used primarily to treat iron toxicity and related diseases,^19^ its use has been rationalized in other areas such as cancer therapy^20−22^ and as a treatment option for parasitic diseases.^23,24^ Recently, a new anticancer drug, mitoDFO, was designed and successfully tested in mouse models. In this compound, DFO is targeted to the mitochondria by two TPP moieties linked by 10-carbon linker chains.^25^ Exposing cancer cell lines to mitoDFO led to its accumulation in mitochondria with consequent iron deficiency in the form of decreased iron–sulfur cluster and heme biogenesis and an overall decrease in the activity of iron-containing enzymes, impaired mitochondrial respiration, increased radical oxygen species production, and the induction of mitophagy. Given the successful recent repurposing of MitoTam as an antiparasitic and antifungal agent,^8^ this study aims to describe the potential of mitochondrially targeted DFO as a selective agent against various important pathogenic eukaryotic microorganisms.

## Results

### Mitochondrially Targeted DFO Inhibits the Growth of Diverse Eukaryotic Unicellular Pathogens

**Figure 1:**
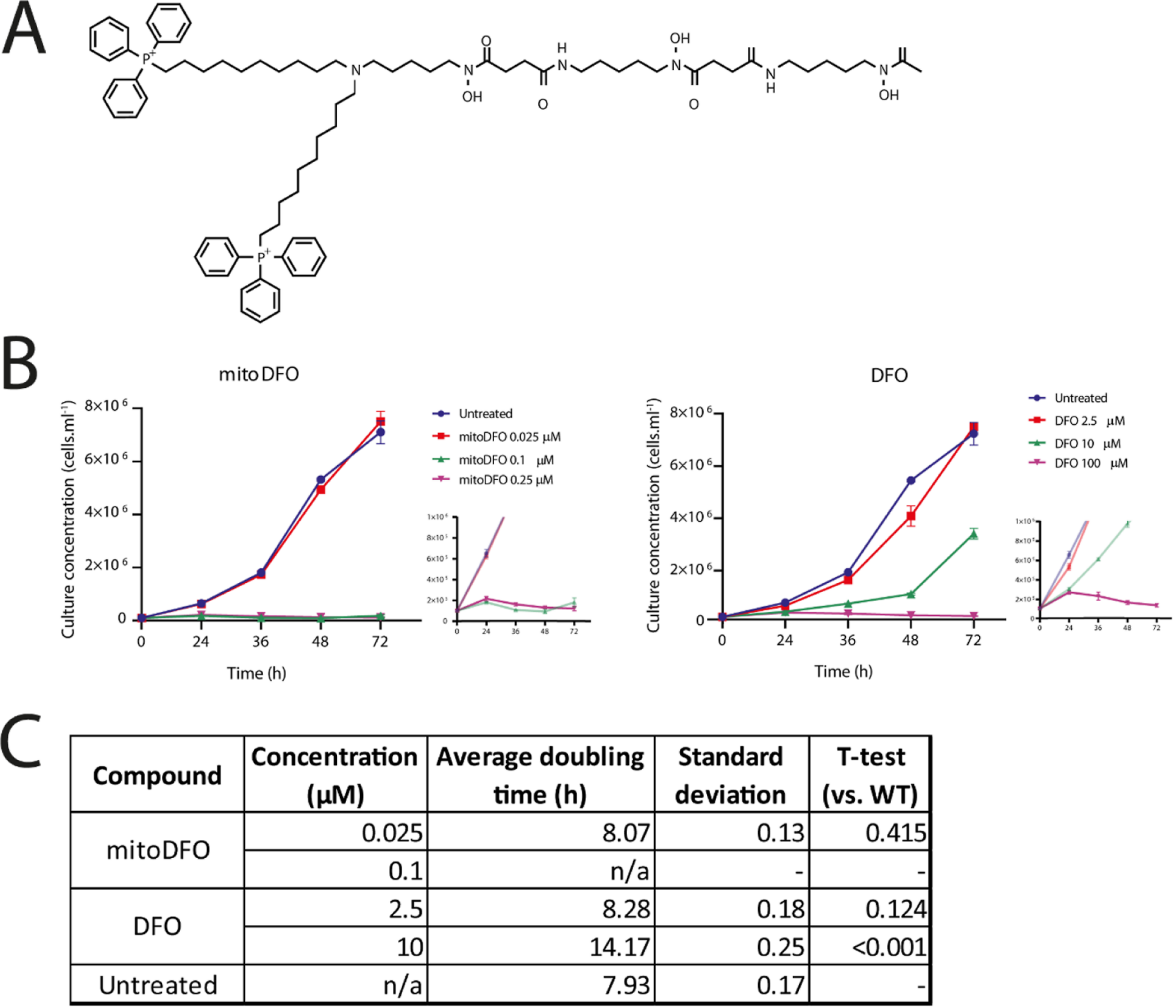
(A) Chemical structure of mitoDFO. (B) Growth curves of *Trypanosoma brucei* 427 bloodstream forms under the effect of mitoDFO and DFO, respectively. Growth was assessed by flow cytometry. Untreated cells (blue) were compared with cells incubated with 0.025 and 0.1 μM mitoDFO or 2.5 and 10 μM DFO, mean values ± s. d. from three biologically independent replicates are given. The inset graph shows the behavior of the culture at higher concentrations of compounds. (C) Calculated doubling times of the growth curves of *T. brucei* 427 bloodstream forms, values are calculated from growth differences between 24 and 48 h.

Since the mitochondrion is the center of iron metabolism, we compared the effect of DFO and its mitochondrially targeted counterpart, mitoDFO (Figure 1A) on a broad spectrum of eukaryotic unicellular pathogens. The results of in vitro screening expressed as half-effective concentrations (EC_50_) are given in Table 1. While DFO did not show substantial efficacy, with the exception of*Trypanosoma gambiense*, the modified chelator mitoDFO showed a significant inhibitory effect against most of the selected parasites. The EC_50_ values of mitoDFO against *Leishmania* amastigotes and promastigotes, *Babesia divergens*, the pathogenic yeast *Cryptococcus neoformans,* and the amphizoic amoeba *Acanthamoeba castellanii* were in the low micromolar concentrations, while for the bloodstream forms of *Trypanosoma brucei* 427 and *T. gambiense* and for *Plasmodium falciparum*, the values were in the nanomolar range (59 ± 22, 181 ± 43, and 334 ± 10 nM, respectively), demonstrating a strong improvement in the compound’s efficacy by tagging it with TPP. Efficacy against *T. brucei* 427 was almost 2 orders of magnitude higher than that of fexinidazole (EC_50_ 4,84 ± 0,28 μM). Human fibroblasts were least affected by the mitochondrially targeted chelator with an EC_50_ value of 8.9 (±3.2) μM, which is consistent with the published value and promises a favorable therapeutic window, particularly for kinetoplastids and *Plasmodium*.^25^*G. intestinalis* was not affected by mitoDFO, most likely due to the absence of conventional energized mitochondria in this microaerophilic parasite, as already observed for MitoTam.^8^ Interestingly, in the two *T. brucei* strains tested, the difference in the efficacy of the two chelators was substantial. We do not have an explanation for this, but it may be related to the fact that strain 427, unlike the highly virulent STIB strain, is a well-established laboratory model that can be adapted to culture conditions, and its iron requirements may be lower, thus reducing its susceptibility to DFO, while its higher susceptibility to mitoDFO may be related to its mitochondrial properties.

**Table 1:** Mean EC_50_ Values for DFO and mitoDFO, Iron-Free (−Fe) or Pre-treated With Equimolar Iron (+Fe), and the Reference Compound^a^

	DFO			mitoDFO			mitoDFO EC_50_ ratio + Fe/–Fe	mitoDFO *t*-test + Fe/–Fe	DFO/mitoDFO ratio (−Fe)	DFO/mitoDFO *t*-test (−Fe)	control	
	–Fe	+Fe	selectivity (for −Fe)	–Fe	+Fe	selectivity (for −Fe)					compound	EC_50_
Human fibroblast BJ1	>100	>100	N/A	8.9 ± 3.2	22.2 ± 0.9	N/A	2.5	0.0005	>11.2	N/A	mitoDFO	7.9 ± 3.9A
T. brucei 427 (BSF)	22.0 ± 1.2	>50	>5	0.059 ± 0.022	0.122 ± 0.038	151	2.1	0.0094	372.9	<0.0001	pentamidine	0.007 ± 0.001
T. brucei STIB (BSF)	2.606 ± 0.753	14.524 ± 1.183	>38	0.320 ± 0.067	0.337 ± 0.042	28	1.2	0.7256	8.1	0.0011	pentamidine	0.001 ± 0.0004
T. gambiense (BSF)	2.471 ± 1.158	>25	>40	0.181 ± 0.043	0.218 ± 0.052	49	1.2	0.2496	13.7	0.0018	pentamidine	0.002 ± 0.0005
L. mexicana (amastigote)	>25	>25	N/A	1.6 ± 0.5	2.4 ± 0.5	6	1.5	0.0238	>15.6	N/A	AmpB	0.320 ± 0.035
L. major (promastigote)	>25	>25	N/A	1.9 ± 0.6	9.2 ± 3.3	5	4.8	0.0017	>13.2	N/A	AmpB	0.024 ± 0.008
B. divergens	10.8 ± 1.3	>50	>9	2.3 ± 0.8	5.8 ± 0.3	4	2.5	0.0000	4.7	<0.0001	atovaquone	0.008 ± 0.001
P. falciparum	12.7 ± 0.6	>50	>8	0.33 ± 0.01	0.45 ± 0.03	27	1.4	0.0052	38.0	<0.0001	chloroquine	0.015 ± 0.001
C. albicans	>100	>100	N/A	3.6 ± 1.7	9.5 ± 1.7	2	2.6	0.0002	>27.8	N/A	AmpB	1.5 ± 0.1
C. neoformans	>100	>100	N/A	2.0 ± 0.5	4.1 ± 1.0	4	2.1	0.0014	>50	N/A	AmpB	0.2 ± 0.1
G. intestinalis	>100	>100	N/A	>50	>50	N/A	N/A	N/A	N/A	N/A	benznidazole	2.6 ± 1.4
N. fowleri	19.5 ± 5.9	>100	>5	6.7 ± 0.7	20.0 ± 2.4	1	3.0	0.0001	2.9	0.0093	AmpB	0.069 ± 0.009
A. castellanii	8.9 ± 0.6	>50	>11	0.8 ± < 0.1	1.0 ± < 0.1	11	1.3	0.0001	11.1	<0.0001	AmpB	8.1 ± 1.6

a Selectivity is compared for iron-free compounds and is calculated as the ratio of average EC_50_ values against a given organism versus the average EC_50_ value against human fibroblasts. The mitoDFO EC_50_ ratio + Fe/–Fe denotes the ratio of average values of iron-free and iron-bound mitoDFO, and the *t*-test for this difference is calculated. All values are from at least three biologically independent replicates. All values are given in μM concentrations. A source C. Sandoval-Acuña et al.^25^

It is expected that pretreatment of either chelator with iron in equimolar concentrations will at least partially neutralize their effects. This is particularly evident in the case of the effect of DFO on *Trypanosoma*, *Babesia*, *Naegleria*, and *Acanthamoeba*, organisms that are relatively susceptible to this chelator, where the addition of iron pushed the EC_50_ values above the assay threshold. However, the addition of iron to mitoDFO resulted in a relatively mild reduction in efficacy in all of the affected organisms (1.2 to 4.8-fold), and in the case of trypanosomes, *P. falciparum*, and *Acanthamoeba*, the EC_50_ values remained in the submicromolar range, suggesting that the effect of this compound is not solely based on iron chelation and that another mechanism(s) underlying its action exists.

Monitoring the growth curves of the bloodstream forms of *T. brucei* 427 (Figure 1B) shows that approximately half the mitoDFO EC_50_ concentration does not affect cell proliferation, while twice the EC_50_ concentration stops its growth. On the other hand, DFO at half the EC_50_ concentration slows down cell proliferation and significantly increases the doubling time (Figure 1C), suggesting a cytostatic rather than cytotoxic mode of action. This apparent effect of DFO occurs at a concentration approximately 100 times higher than that of mitoDFO, further illustrating the marked efficacy of the latter compound. Nevertheless, the cytostatic mode of action is also prevalent for mitoDFO, since after 24 h of incubation with up to 0.25 μM concentration of the mitochondrial chelator, more than 90% of the cells were alive, and incubation with twice the EC_50_ concentration resulted in more than 95% living cells even after 36 h of incubation, as shown in Table S1.

### MitoDFO Affects the Biochemical and Physicochemical Properties of Mitochondrion in Bloodstream Forms of *T. brucei* 427

**Figure 2:**
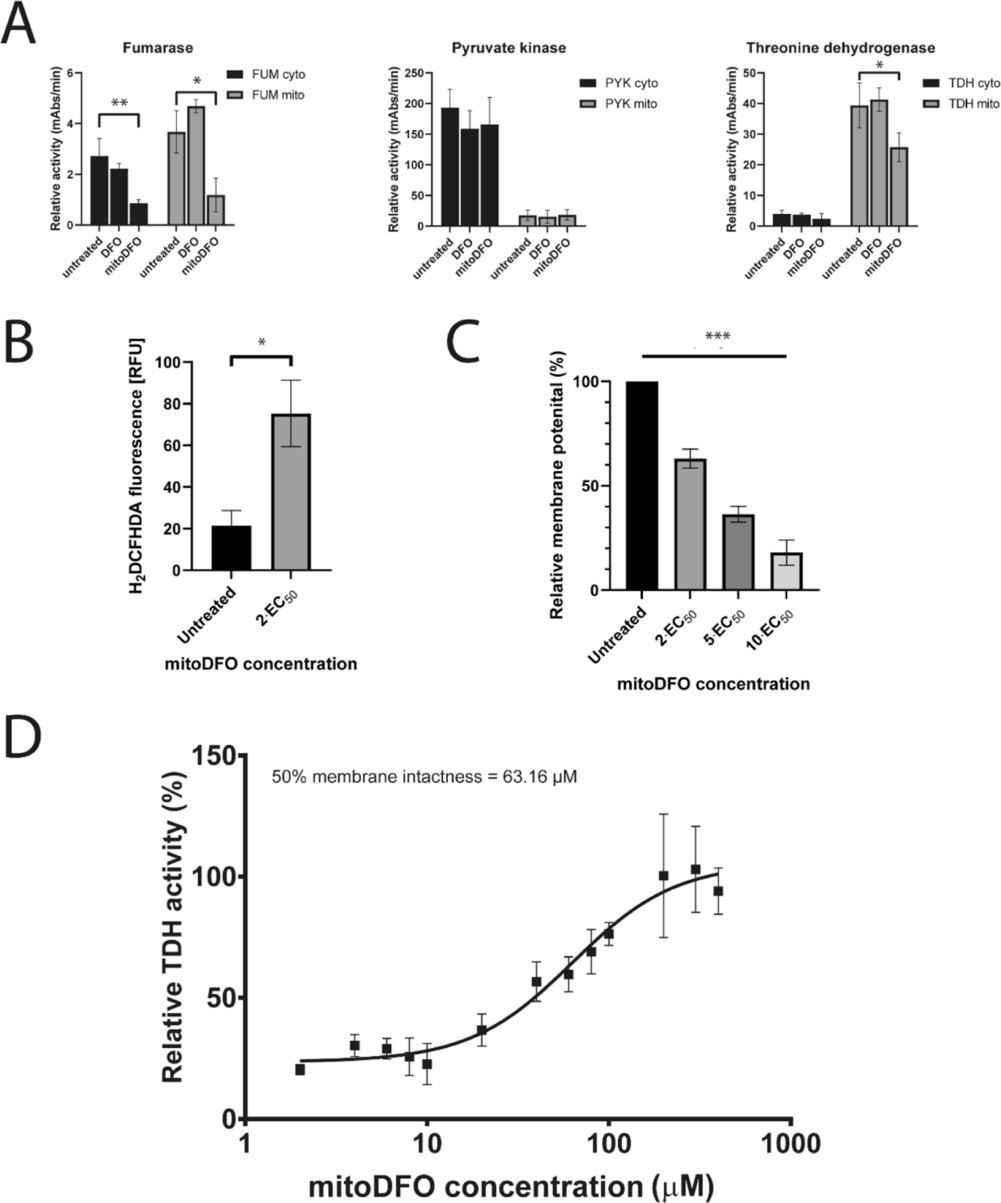
Effect of mitoDFO on *Trypanosoma brucei* 427 bloodstream form cells. (A) Effect of 0.12 μM DFO and mitoDFO exposure for 24 h on the enzymatic activity of cytosolic and mitochondrial iron-containing fumarase, cytosolic pyruvate kinase, and mitochondrial threonine dehydrogenase. Values are given as the mean activities of three biologically independent replicates ± s. d. Stars denote statistically significant changes *: *P* < 0.05 and **: *P* < 0.01. (B) Production of oxygen radicals, quantified by flow cytometry using the H_2_DCFHDA detection kit in untreated cells and cells treated with 0.12 μM mitoDFO for 24 h. Values are given as mean ± s. d. Star denotes statistically significant changes *: *P* < 0.05. (C) Mitochondrial membrane potential of *T. brucei* 427 under the effects of 0.12, 0.3, and 1.2 μM mitoDFO for 24 h. Values are given as mean ± s. d. The star denotes a statistically significant trend calculated by RM one-way ANOVA with a Geisser-Greenhouse correction. ***: *P* < 0.001. (D) Mitochondrial membrane intactness was assessed in isolated mitochondria under the effects of different concentrations of mitoDFO. Mitochondrial threonine dehydrogenase activity was detected as a marker for membrane permeability, as it can be detected only if the membrane is compromised to such an extent that the substrates can diffuse freely.

To investigate the antiparasitic effect of mitoDFO, we focused on bloodstream forms of *T. brucei*427. This organism possesses a single mitochondrion per cell with some distinguishing characteristics and is a widely recognized and well-studied model organism for in vitro and in vivo assays. The iron-chelating potential of mitoDFO in the cell was investigated by assessing the activity of iron-containing fumarase, an enzyme located in both the cytosol and the mitochondrion of this organism. The concentration of mitoDFO used was twice the value of EC_50_ with exposure of cells for 24 h. To compare the effects of the two chelators, we used the same concentration of DFO. As can be seen in Figure 2A, treatment with mitoDFO significantly reduced both cytosolic and mitochondrial fumarase activity (each approximately 3-fold), while poorly membrane-permeable DFO at the same concentration had no significant effect. Pyruvate kinase, which is located in the cytosol of *T. brucei* and does not contain iron as a cofactor, showed no difference in activity due to the effect of chelators, whereas threonine dehydrogenase, also a noniron enzyme located in the mitochondrion, was significantly reduced by mitoDFO but not DFO (Figure 2A).

Reactive oxygen species (ROS) are highly reactive compounds that are commonly produced by redox reactions. Beneficial contributions of ROS to cellular processes have been described; however, they are stress markers that cause cellular damage at abnormal levels. Compounds containing TPP were shown to significantly increase ROS production;^9^ therefore, it was reasonable to expect the same result with mitoDFO. Incubation of *T. brucei* 427 culture with twice the EC_50_ value of mitoDFO for 24 h caused a significant increase in the presence of ROS (*t*-test *P*-value < 0.05), approximately 3.5-fold compared to the culture without treatment (Figure 2B). Although the mitochondrial function of *T. brucei* differs fundamentally from the classical model of this organelle, the mitochondrial membrane potential of the parasite is meticulously maintained by reversed F_O_F_1_ ATPase.^26^ This crucial feature of mitochondria is rapidly reduced in a dose-dependent manner by mitoDFO (ANOVA *P*-value < 0.001), as shown in Figure 2C. We have previously demonstrated that mitochondrially targeted tamoxifen directly disrupts the integrity of the IMM in *T. brucei* 427.^8^ Using threonine dehydrogenase activity as a marker for the membrane permeability of isolated mitochondria, we observed a similar effect of mitoDFO (Figure 2D). Treatment with 63 μM mitoDFO increased inner membrane permeability by 50%, a milder effect than that of MitoTam, which should nevertheless be considered as one of the possible mechanisms of action of the compound. As with MitoTam, the high concentrations of mitoDFO required for this observation are probably due to the absence of the dramatic mitochondrial accumulation of phosphonium salts that occurs in living cells.

### MitoDFO has the Potential for the Treatment of Intracellular *Leishmania* Infections

The amastigote stage of *Leishmania* parasites resides inside macrophages and causes clinical symptoms of varying severity. This parasite effectively exploits the immune system for its propagation while evading the host response. The challenging task of delivering an effective compound to the mitochondria of intracellular parasites, hiding behind several biological membranes, could be solved using lipophilic cations such as TPP-based compounds. They are distributed based on membrane potential and have been shown to be efficiently transported to amastigotes, residing in the acidic environment of the phagolysosome.^27^ To investigate how effective mitoDFO is against intracellular *Leishmania* infection, we infected mouse macrophages with GFP-expressing *Leishmania mexicana* and subjected the infected macrophages to a three-day treatment. Taking advantage of the GFP-tagged parasite, we quantified the extent of infection by flow cytometry.

**Figure 3:**
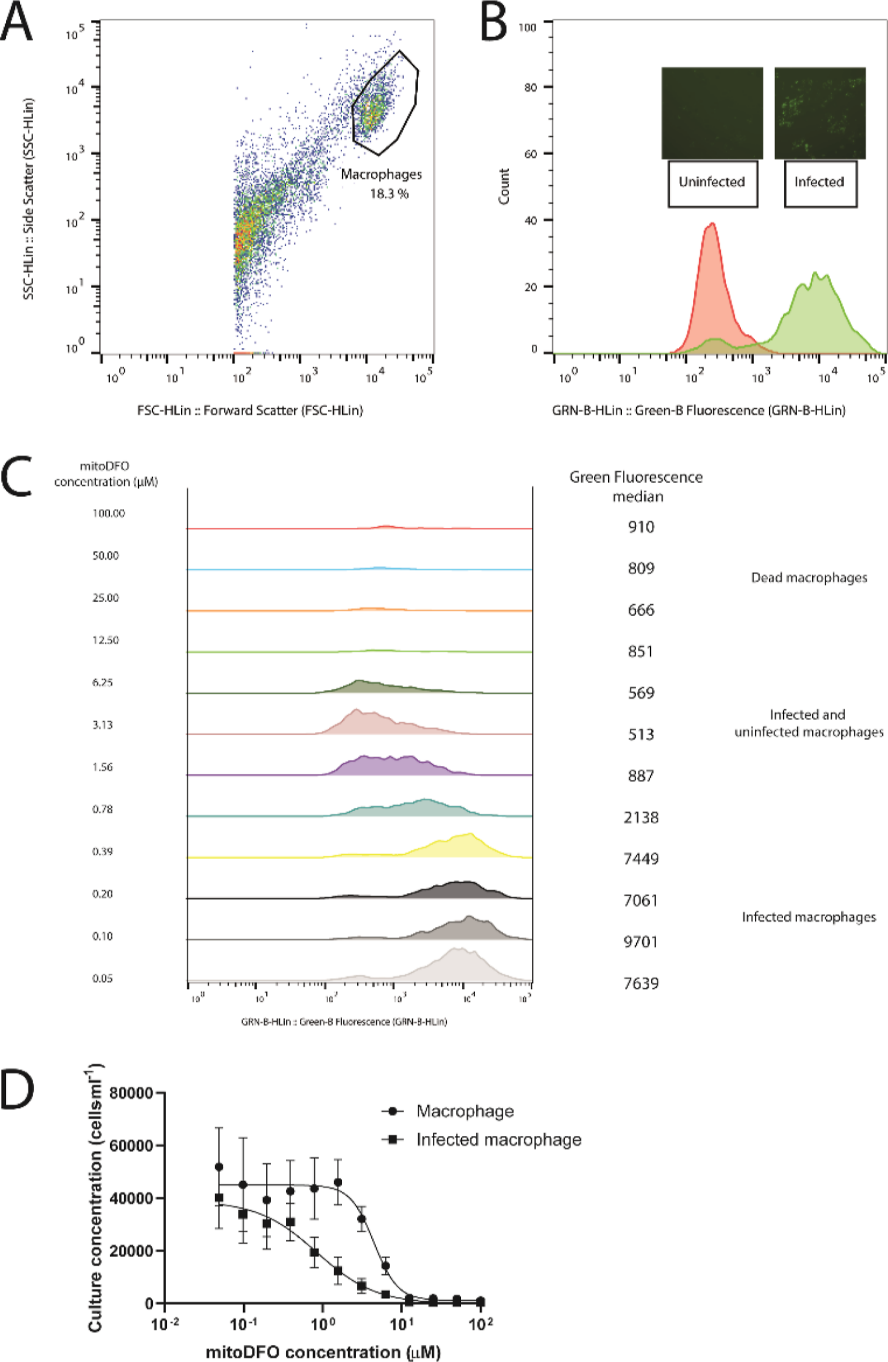
Intracellular *Leishmania mexicana* drug sensitivity assay. (A) Mouse macrophage population was differentiated using flow cytometry. (B) Subsequently, uninfected macrophages were differentiated from macrophages infected by *Leishmania mexicana* amastigotes expressing GFP using 488 nm excitation and a 525/30 nm detector. (C) Range of concentrations of mitoDFO vary in their effect on the infected culture, as shown by a shift in the median fluorescence. (D) From this data, EC_50_ curves were derived, showing the effect of mitoDFO concentration on the total population of macrophages and the portion of infected macrophages.

Figure 3 shows that the macrophage population differentiates into two populations with different fluorescence intensities corresponding to the GFP signal (B). Under increasing concentrations of mitoDFO, it is apparent that the intensity of fluorescence decreases, suggesting that the parasite population is decimated and the infection load on the macrophage is decreasing (C). This method allowed us to determine the EC_50_ values for intracellular parasites. As summarized in Table 2, mitoDFO was more effective against intracellular amastigotes than against axenic parasites (a 2-fold decrease in EC_50_ concentration), indicating that the compound is able to specifically target the phagolysosome. In contrast, amphotericin B, a commonly used antiparasitic agent, showed lower efficacy against intracellular amastigotes.

**Table 2:** EC_50_ Values of Tested Compounds for Cultures of Murine Macrophage and *Leishmania mexicana* Axenic Form in Axenic Monoculture and Infection Assay^a^

	axenic		infection assay	
	murine macrophage	Leishmania mexicana	murine macrophage	Leishmania mexicana
mitoDFO	6.34 ± 4.45	1.56 ± 0.50	4.67 ± 1.18	0.67 ± 0.24
DFO	>25	>25	45.27 ± 3.09	47.05 ± 2.91
amphotericin B	>25	0.33 ± 0.08	29.09 ± 5.91	3.77 ± 0.40
				
MitoTam	0.39	0.35	0.26	∼0.1

a Values are given as mean ± s. d. from at least three biologically independent plicates.

### Pathogenic Yeast Virulence Factors are Influenced by mitoDFO

**Figure 4:**
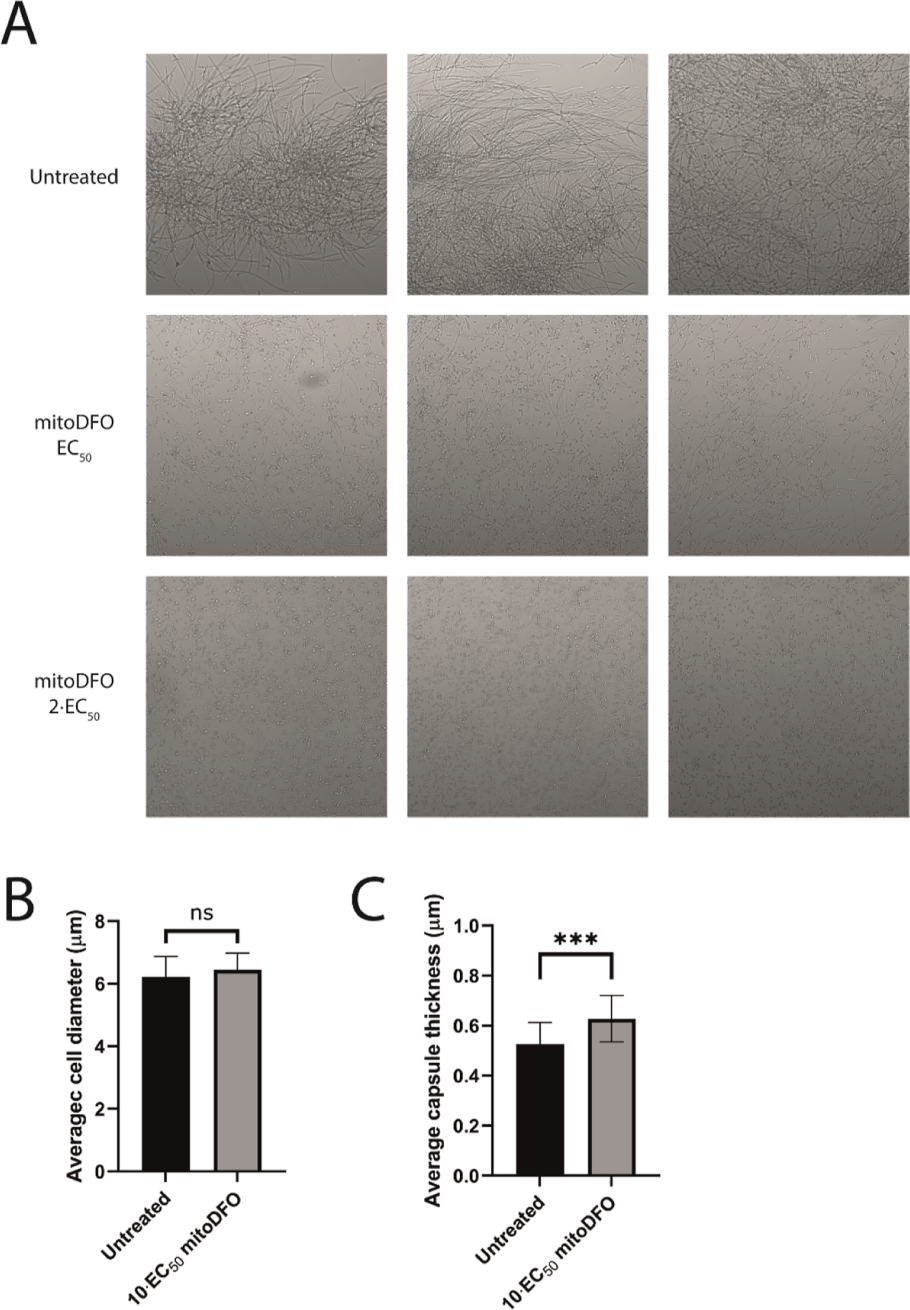
Effect of mitoDFO on pathogenic yeasts. (A) *Candida albicans* culture treated with 3.6 and 7.2 μM mitoDFO for 24 h. In comparison to the untreated culture, an absence of hyphal growth can be seen in the treated cultures. (B) *Cryptococcus neoformans* average cell diameter when treated with 20 μM mitoDFO for 24 h. Randomly, 31 cells were measured. (C) In the same assay, average capsule thickness was measured, ***: *P* < 0.001.

The fungal pathogen *Candida albicans* can exist in different morphological appearances, including typical yeast coccus and multicellular hyphal form, which is related to its ability to cause candidiasis.^28,29^ Although this work shows that DFO has a small effect on the growth of the culture, mitoDFO had a notable inhibitory effect on yeast viability (Table 1). Therefore, we attempted to assess the effect of mitoDFO on the morphology of the culture. *C. albicans* grown under the influence of the compound rapidly loses its ability to grow in the invasive forms of pseudohyphae or hyphae, as can be seen in Figure 4A. Another yeast pathogen, *C. neoformans*, possesses a distinct cell capsule, which is an important virulence factor,^30−32^ and its thickness is known to be affected by external stimuli.^33^ Exposure of *C. neoformans* to mitoDFO and staining it using ink followed by microscopy and in silico measurement of single yeast cells confirmed that the thickness of this capsule increases significantly under the influence of mitoDFO, while the overall cell size remains the same compared to the untreated culture (Figure 2B,C). Thus, *C. neoformans* may respond to iron deprivation by thickening its protective capsule.

## Discussion

Therapeutic intervention via targeting the mitochondria of pathogenic microorganisms and cancer cells is an attractive and promising approach due to the metabolic differences between normal and cancer cells as well as between host and pathogen. The combination of a mitochondrial targeting vector with an iron chelating molecule was previously proposed in the area of tumor biology.^25^ Our investigation of the effect of a novel compound, mitoDFO, and its parent compound, DFO, on a spectrum of eukaryotic pathogenic microorganisms revealed several key findings: (i) mitoDFO is more effective in inhibiting the selected parasites compared to DFO, (ii) compared to hosts cells, mitoDFO exhibits significant selectivity for *Trypanosoma* and *P. falciparum*, (iii) the addition of extracellular iron renders DFO ineffective, while the effect of mitoDFO is only moderately reduced, (iv) due to its chemical properties and electrochemical gradient across the macrophage and the membranes of the intracellular parasite, mitoDFO is more effective against intracellular *Leishmania* parasites, and (v) there is no obvious difference in susceptibility to these two chelators between strictly host-dependent parasites and free-living opportunistic parasites, which might be expected to have different iron homeostasis strategies. This is evident in the case of *A. castellanii*, which must adapt to constant changes in iron availability and is more sensitive to mitoDFO than *Babesia*, which resides in the relatively stable environment of the erythrocyte. Thus, the selectivity of mitoDFO appears to involve a more complex mechanism than simply the requirement for iron and the efficiency of its acquisition. These results reveal the unique potential of mitoDFO as a novel antiparasitic compound. In addition, our data indicates an additional mode of action besides iron chelation in mitochondria in some pathogens. For example, in *P. falciparum*, excess iron did not affect mitoDFO’s EC_50_, and this compound may act via the TPP vector and/or the linker.

Employing *T. brucei* 427 as an established model to study mitochondrial processes, we showed that the enzymatic activities of iron-containing fumarase were significantly reduced in both the cytosolic and mitochondrial fractions under the influence of mitoDFO, indicating iron deprivation. Consistent with the dramatically lower effect of DFO on trypanosome growth, the equal concentration of this chelator did not cause differences in fumarase activity. As with the overall effect on the parasite, this is due to the difference in delivery to the cell; DFO is poorly permeable across cellular membranes and enters cells by endocytosis,^34^ whereas mitoDFO is trafficked and accumulated in the cell proportionally to the membrane potential as described for lipophilic cations.^1^ While the activity of pyruvate kinase, a cytosolic enzyme without an iron cofactor, was not altered by mitoDFO, the activity of mitochondrial threonine dehydrogenase was significantly reduced by this chelator. The effect on this enzyme, which is not iron-dependent, must again be explained by mechanisms other than chelating activity. It has long been hypothesized that lipophilic cations integrate into the IMM and accumulate there, interfering with membrane integrity and nonspecifically inhibiting mitochondrial enzymes, and our study supports this conclusion.^1^ Furthermore, we have observed the direct effect of mitoDFO on the integrity of the IMM at micromolar concentrations, analogously to that of mitochondrially targeted tamoxifen.^8^

One of the relevant indicators of mitochondrial interference is the increased presence of free radicals, which we observed in *T. brucei* 427 upon exposure to mitoDFO. ROS levels can become dysregulated as a result of external stimuli, such as mitochondrial damage, or by inhibition of ROS-scavenging enzymes, such as superoxide dismutases, some of which are known to be iron-dependent in trypanosomatids.^35^ The exact mechanism of ROS generation by mitoDFO is not completely clear yet. While reduced availability of iron for ROS-scavenging enzymes may be one of the underlying factors, overall damage to mitochondria also leads to ROS formation, and the production of ROS by phosphonium salts has been shown in *Leishmania*.^9^ Together, these results suggest that mitoDFO is trafficked into the mitochondrion, where DFO chelates iron and causes iron deprivation both in the organelle and, mainly due to the defect in iron–sulfur cluster biogenesis, in the whole cell. This, together with direct disruption of the integrity of the IMM, impairs mitochondrial function and affects whole-cell metabolism and cell viability.

The selectivity of mitoDFO against pathogenic yeasts was less pronounced than against trypanosomes, *A. castellanii*, or *P. falciparum*. However, the incidence of infections caused by pathogenic yeasts combined with the difficulties in treating systematic infections^36^ and emerging drug resistance^37,38^ calls for the repurposing of current chemotherapeutics or the discovery of novel compounds, and mitochondrially targeted chelators might represent new antifungal candidates. Notably, we have shown that mitoDFO interferes with the virulence factors of two fungal pathogens, i.e., *C. albicans* and *C. neoformans*. A prominent morphological change was observed in *C. albicans*, where the chelator prevented the formation of hyphae or pseudohyphae, which are prerequisites for candidiasis.^28,29^ Exposure of *C. neoformans* to mitoDFO led to an increase in the cell capsule size. The capsule plays an important role in *C. neoformans* biology, including its protection against unfavorable conditions.^30^ Interestingly, iron depletion has been shown to induce the formation of *C. neoformans* capsules,^39^ which have low nutrient levels.^33^ Whether this effect is related to iron chelation or due to overall toxicity remains to be investigated.

Although less effective than against most other tested species, our study demonstrates effectiveness at low micromolar concentrations against RBC-cultured *B. divergens*. Our findings can be directly compared with previously reported results on the effect of DFO against *Babesia gibsoni*.^40^ The effect of DFO on *B. divergens* was slightly lower, with EC_50_ values of 10.8 ± 1.3 μM, compared to 6.45 ± 3.43 μM in *B. gibsoni*. Nevertheless, mitoDFO compounds exhibit several-fold improved efficacy compared to DFO and should be considered novel compounds for the development of specific chemotherapy for *Babesia*/*Theileria* infections.

In conclusion, this work presents mitoDFO, a compound originally developed for use in cancer therapy, as a promising alternative for the treatment of a range of parasitic diseases, including those caused by intracellular parasites *Plasmodium* and *Leishmania*, due to its unique combination of iron-chelating properties and mitochondrial trafficking and damage. Future research should focus on optimizing mitochondrial chelators as antiparasitic agents by testing chelators with different chelating properties and/or lipophilicity, as well as by modifying the mitochondrial targeting moiety, where changes in mitochondrial accumulation due to variations in both TPP and linkers would affect biological activity.

## Methods

### Cultivation and Drug Sensitivity Assays

All organisms were cultivated according to the conditions summarized in Table S2, which also includes strain specifications. Dose–response curves were obtained by their cultivation on 96 well plates in a 2-fold series dilution of the appropriate drug, in a total volume of 200 μL (with the exception of *P. falciparum*, where the total volume was 100 μL). Iron supplementation was achieved by mixing the compound with Fe-NTA in an equimolar ratio prior to setting up the experiment. Inoculum concentrations, incubation times, and methods of assessment of culture viability are summarized in Table S2.^41−44^ Results were plotted and processed using Prism 8.0 (GraphPad Software). A two-tailed unpaired *t*-test was performed to assess the significance of the difference between mitoDFO with and without added iron. Selectivity was calculated as the ratio of the average EC_50_ value for human fibroblasts to the average EC_50_ value for the appropriate pathogen. All data were obtained from a minimum of three independent biological replicates.

### *T. brucei* Growth Curves

Bloodstream *T. brucei* 427 cells were inoculated to a concentration of 1 × 10^6^ cells per ml and incubated with the addition of mitoDFO (0.025 and 0.1 μM) or DFO (2.5 and 10 μM) in aerobic flasks in a total volume of 5 mL in 5% CO_2_ at 37 °C. Control with no compound was used to obtain reference values. At time points 24, 36, 48, and 72 h, a 20 μL sample was collected, diluted in growth medium, and measured on a Guava EasyCyte 8HT flow cytometer (Luminex) to assess concentration in previously experimentally prepared settings. The culture was simultaneously observed by light microscopy to confirm the cell viability. The growth curve was plotted by using Prism 8.0 (GraphPad Software). All conditions were measured in three biologically independent replicates. Doubling time was calculated between 24 and 48 h time points using the online calculator,^45^ and each of the conditions was compared to the untreated culture using a two-tailed unpaired *t*-test.

### *T. brucei* Enzymatic Assays

*T. brucei* 427 bloodstream forms were preincubated for 24 h in appropriate conditions (0.12 μM mitoDFO, 0.12 μM DFO, and no addition) and harvested, and cytosolic and mitochondrial fractions were separated by digitonin fractionation as described previously.^46^ Briefly, cells were spun down (1200 g, 15 min, 4 °C) and resuspended in SHE buffer (250 mM sucrose, 25 mM HEPES, 1 mM EDTA, pH 7.4), and protein concentration was measured using a BCA kit (Sigma-Aldrich). Subsequently, cells were transferred into HBSS (Sigma-Aldrich) and digitonin (Calbiochem) was added in the protein/digitonin ratio of 1/0.15 for 4 min and spun (21 000 g, 2 min, 4 °C). The supernatant was collected and placed on ice as a cytosolic fraction. The remaining pellet was lysed using 0.1% Triton X-100 in HBSS for 5 min, resuspended, washed in HBSS twice, and used as a mitochondrial fraction. The activities of cytosolic pyruvate kinase and mitochondrial threonine dehydrogenase were assessed in all conditions using the spectrophotometric assay at 340 nM, as described in ref (46), and served both as markers of successful fraction preparation and to assess the effect of the studied compounds. Briefly, pyruvate kinase was measured in TEA buffer (0.1 M triethanolamine, 5 mM MgSO_4_, 50 mM KCl, pH 7.6) with added 2.8 mM phosphoenolpyruvate, 2 mM ADP, 0.3 mM NADH, and lactate dehydrogenase. Threonine dehydrogenase was measured in 0.2 M Tris–HCl buffer, pH 8.6, 0.25 KCl with 120 mM threonine, and 2.5 mM NAD^+^. Fumarase was measured at 240 nM in 2 mM Tris–HCl buffer, pH 7.5, with 20 mM malate.

Mitochondrial intactness was assessed by isolating the mitochondrial fraction of untreated cells, as described above, without the addition of Triton X-100 and adding different concentrations of mitoDFO. Threonine dehydrogenase activity was measured as a marker of the disintegration of mitochondrial membranes. Triton X-100 was used to disrupt membrane integrity and served as a positive control to obtain maximum activity. All experiments were performed in at least three biologically independent replicates and plotted and analyzed using Prism 8.0 (GraphPad Software).

### Reactive Oxygen Species Determination in *T. brucei*

*T. brucei* 427 bloodstream form’s cellular ROS production was assessed using H_2_DCFDA (Sigma-Aldrich). Cells were preincubated for 24 h with 0.12 μM mitoDFO, and approximately 1 × 10^6^ cells were incubated with 10 μM H_2_DCFDA for 30 min and washed with PBS with 6 mM glucose, and 10 000 events were measured on a Guava EasyCyte 8HT flow cytometer (Luminex) using a 488 excitation laser and a 525/30 detector. Median fluorescence was plotted against untreated culture using Prism 8.0 (GraphPad Software), and statistical significance was determined using a two-tailed paired *t*-test. The experiment was performed in three biologically independent replicates.

### Mitochondrial Membrane Potential in *T. brucei*

*T. brucei* 427 bloodstream form’s mitochondrial membrane potential was assessed using the fluorescent probe TMRE (Thermo Fisher Scientific). Cells were preincubated for 24 h with 0.12, 0.30, and 0.59 μM mitoDFO, and a negative control was obtained using untreated cells with 20 μM FCCP uncoupler. Approximately 1 × 10^6^ cells were incubated with 60 nM TMRE for 30 min and washed with PBS with 6 mM glucose, and 10 000 events were measured on a Guava EasyCyte 8HT flow cytometer (Luminex) using a 488 excitation laser and a 583/26 detector. Median fluorescence was plotted against untreated culture using Prism 8.0 (GraphPad Software), and statistical significance was determined using RM one-way ANOVA with Geisser-Greenhouse correction. The experiment was performed in three biologically independent replicates.

### Intracellular *L. mexicana* Macrophage Infection

Approximately 10 000 murine macrophages per well were seeded on 96 well plate, and a suspension of 60 000 *L. mexicana* GFP-expressing promastigotes was added to each well and left to infect the mammal cells for 72 h in a cultivation medium under the conditions described in Table S2. 2-fold dilution series starting with 100 μM of either mitoDFO, DFO, or Amphotericin B in a regular growth medium were added, and cultures were incubated for further 72 h in the same conditions. Macrophage cells were washed and resuspended in growth medium and detached by pipetting, and their concentration in each well was measured on a Guava EasyCyte 8HT flow cytometer (Luminex). The GFP signal of internalized *L. mexicana* amastigotes in each macrophage was detected using a 488 nm excitation laser and a 525/30 nm detector. Median fluorescence graphs were plotted, and EC_50_ values for both macrophages and intracellular *L. mexicana* were calculated using Prism 8.0 (GraphPad Software).

### *C. albicans* Hyphae Formation and *C. neoformans* Capsule Size

The yeast form of *C. albicans* was seeded in the 2 mL RPMI medium on a 24 well plate with a glass bottom and treated with MitoDFO in concentrations of 0, 3.6, and 7.2 μM. The culture was incubated at 35 °C for 24 h, and microscopic images were taken using phase contrast on an inverted microscope Eclipse TI-S (Nikon).

*C. neoformans* capsule thickness was measured using a previously published protocol.^33^ Briefly, the yeast was seeded in 1 mL of RPMI in a 24 well plate with and without a final concentration of 20 μM mitoDFO for 24 h at 35 °C. The culture was harvested (1300 g, 5 min, RT) and resuspended in 50 μL of RPMI. An equal amount of India Ink (Thermo-Fisher) was added, and a total volume of 15 μL of resuspended cells was placed on a microscope slide, covered with cover glass, and imaged on an Eclipse TI-S (Nikon) inverted microscope using 100× magnification. A total of 31 cells were randomly selected, excluding morphologically anomalous and overlapping cells. Using NIS Elements BR (Nikon), the inner and outer diameters of each cell were measured, and cell size and capsule thickness were calculated. Graphs were plotted, and an unpaired *t*-test was performed using Prism 8.0 (GraphPad Software).

### Statistical Analysis

Unless stated otherwise, Prism 8.0 (GraphPad Software) was used to identify outliers using the ROUT method (*Q* = 1%). Statistical significance is displayed on the graph as *: *P* < 0.05, **: *P* < 0.01, and ***: *P* < 0.001.

## Supporting Information Available

Summarization of all cultivated organisms, including strain specifications, cultivation conditions, and the method of assessment of culture viability (XLSX)
Dose–response curves of mitoDFO without iron supplementation for all tested organisms and determination of live and dead treated and untreated cells at selected time points (PDF)
