# Supplementary material for: Chelation of Mitochondrial Iron as an Antiparasitic Strategy
Source: ACS Infect Dis. 2024 Jan 30;10(2):676–87. doi: 10.1021/acsinfecdis.3c00529 (PMC10862539; doi:10.1021/acsinfecdis.3c00529)
Supplement: Supplementary file 2 — id3c00529_si_002.pdf [file id3c00529_si_002.pdf]

Supporting Information

Title: Chelation of mitochondrial iron as an antiparasitic strategy

List of authors:

Dominik Arbon - Department of Parasitology, Faculty of Science, Charles University, BIOCEV, Vestec, 25250, Czech Republic

Jan Mach - Department of Parasitology, Faculty of Science, Charles University, BIOCEV, Vestec, 25250, Czech Republic

Aneta Čadková - Department of Parasitology, Faculty of Science, Charles University, BIOCEV, Vestec, 25250, Czech Republic

Anna Sipkova - Department of Parasitology, Faculty of Science, Charles University, BIOCEV, Vestec, 25250, Czech Republic

Jan Stursa - Institute of Biotechnology, Czech Academy of Sciences, BIOCEV, Vestec, 25250, Czech Republic
- Laboratory of Clinical Pathophysiology, Diabetes Centre, Institute for Clinical and Experimental Medicine, Videnska 1958/9, 140 21, Prague, Czech Republic

Kristýna Klanicová - Institute of Biotechnology, Czech Academy of Sciences, BIOCEV, Vestec, 25250, Czech Republic
- Department of organic chemistry, Faculty of Science, Charles University, Prague, 128 00, Czech Republic

Marta Machado - Graduate Program in Areas of Basic and Applied Biology, Instituto de Ciências Biomédicas Abel Salazar, Universidade do Porto, 4050-313, Portugal
- Centre for Infectious Diseases, Parasitology, Heidelberg University Hospital, Heidelberg, 69120, Germany

Markus Ganter - Centre for Infectious Diseases, Parasitology, Heidelberg University Hospital, Heidelberg, 69120, Germany

Viktoriya Levytska - Institute of Parasitology, Biology Centre, Academy of Sciences of the Czech Republic, Branišovská 1160/31, České Budějovice, 37005, Czech Republic

Daniel Sojka - Institute of Parasitology, Biology Centre, Academy of Sciences of the Czech Republic, Branišovská 1160/31, České Budějovice, 37005, Czech Republic

Jaroslav Truksa - Institute of Biotechnology, Czech Academy of Sciences, BIOCEV, Vestec, 25250, Czech Republic

Lukáš Werner - Institute of Biotechnology, Czech Academy of Sciences, BIOCEV, Vestec, 25250, Czech Republic
- Laboratory of Clinical Pathophysiology, Diabetes Centre, Institute for Clinical and Experimental Medicine, Videnska 1958/9, 140 21, Prague, Czech Republic

Robert Sutak - Department of Parasitology, Faculty of Science, Charles University, BIOCEV, Vestec, 25250, Czech Republic

Corresponding authors: sutak@natur.cuni.cz, Lukas.Werner@ibt.cas.cz

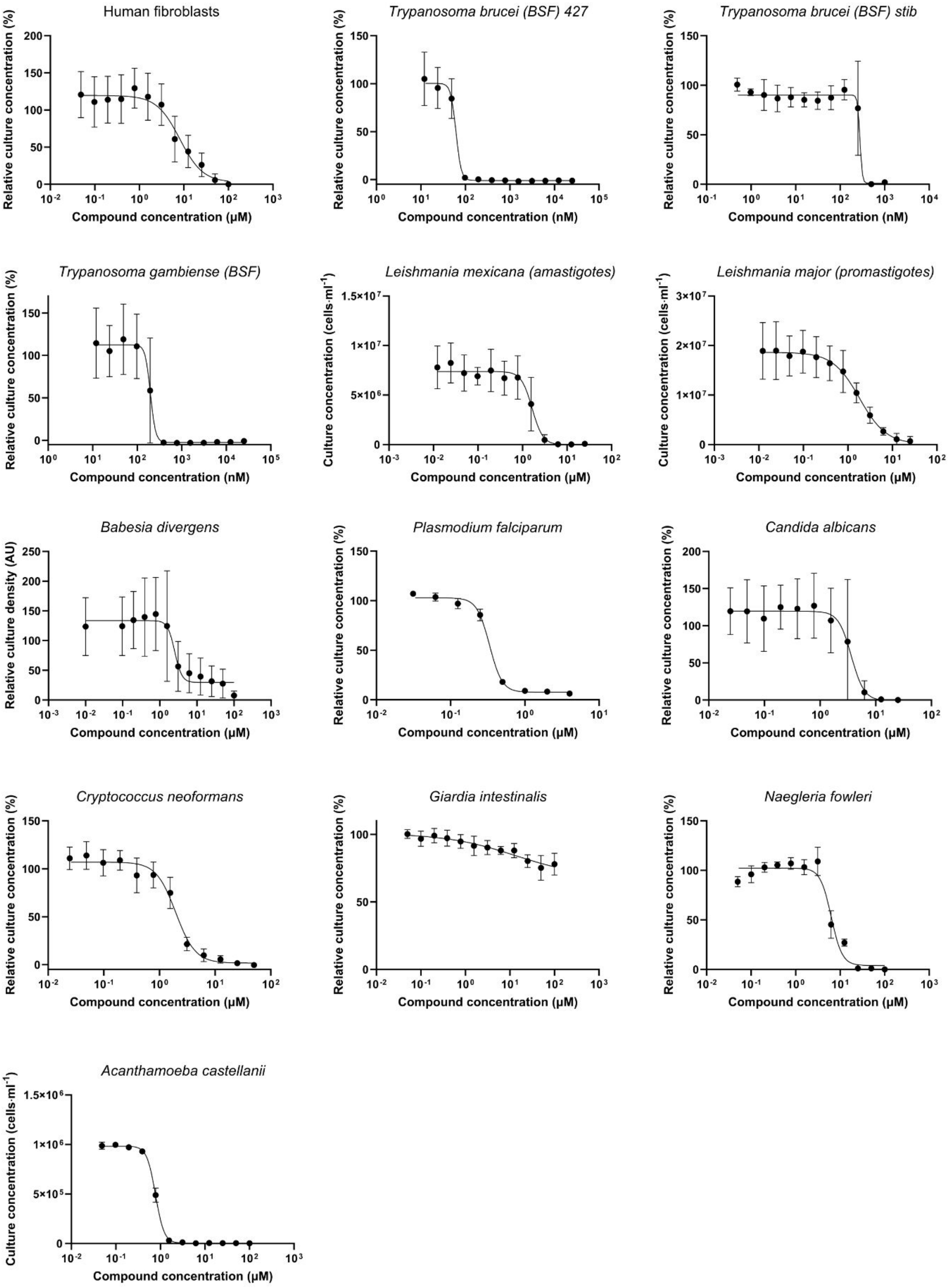

Figure S1

Dose-response curves of mitoDFO without iron supplementation for all tested organisms in Table 1. In vitro assays were performed according to the cultivation conditions and assays described in the methods section and in Supplementary Table 1. Derived EC50 values are summarized in Table 1. The X-axis is in logarithmic scale. Curves were constructed and EC50 values derived using Prism 8.0 (GraphPad software)

Table S1

Sytox Green Assay to determine the percentage of live treated and untreated cells at selected time points. *T. brucei* BSF cells were seeded at a concentration of 2 × 10^5 cells/mL in 5 mL HMI-9 medium in aerobic cultivation flasks. Cells were treated with compounds at final concentration of 0,05 µM, 0,1 µM, 0,25 µM mitoDFO and 10 µM, 100 µM DFO. In addition, there was a group of untreated cells. At defined time points (24, 36, 48, and 72 h), 20 µL samples were collected from each flask. These samples were then diluted tenfold in fresh HMI-9 medium, reaching a final volume of 200 µL, with the addition of 2 µL SYTOX Green Dead Cell Stain (Invitrogen). Following a 30-minute incubation under standard growth conditions, the cells were analyzed using a Guava EasyCyte 8HT flow cyometer (Luminex flow) with detection at 504/523 nm. This entire procedure was conducted in biological triplicates.

**Compound**	**Concentration (µM)**	**% of live cells**			
		24h	36h	48h	72h
mitoDFO	0,05	98,6	96,1	62,1	52,8
	0,1	99,1	97,9	66,2	53,8
	0,25	94,4	82,5	70,2	64,3
DFO	10	99,1	97,5	74,3	58,6
	100	95,5	78,2	62,1	54,8
Untreated	n/a	99	90,8	62	53,2

Table S2

Summarization of all cultivated organisms including strain specifications, cultivation conditions and method of assessment of culture viability.
